# Can global health opportunities lead to an increase in primary care physicians?

**DOI:** 10.7189/jogh.10.020387

**Published:** 2020-12

**Authors:** Josephine Reece, Christopher Dionne, Troy Krupica, Nathan Lerfald, Jenna Sizemore, Sarah Sofka

**Affiliations:** West Virginia University School of Medicine Department of Medicine, Morgantown, West Virginia, USA

Global health opportunities remain popular among United States (US) medical students. According to the annual AAMC (Association of American Medical Colleges) medical student questionnaire, thirty percent of graduating medical students participated in a global health elective during their training [1]. Although this average has remained constant since 2012, the percentage of first year US medical students who plan to participate ranges from sixty to eighty percent. Interest in global health persists even after graduation with 66% of newly graduated physicians planning to participate in global health activities [2]. In addition to experiencing unfamiliar diverse disease pathology, benefits of participating in global health electives includes increased medical knowledge with improved physical exam skills while decreasing reliance on laboratory or radiology studies. Physicians report international health exposure as a factor in fostering cultural sensitivity and awareness, as well as a driving force in pursuing primary care careers [3-5].^5^

There is an increased demand for primary care physicians with shortages in primary care predominantly affecting impoverished people, immigrants or refugees and rural America [1,6]. A study commissioned by the AAMC and updated in 2019 projects that by 2032, there will be a shortage of primary care physicians by a range of 21 000 to 55 000 physicians. The US population 65 and over is expected to expand by almost 50% by 2032 which highlights the urgent demand for more primary care providers [7].

Trainees that take part in global health experiences are more likely to practice primary care with underserved populations. Comparing graduates from an internal medicine (IM) residency program, those that were in an international health program more often changed career paths from subspecialty to general medicine (56% vs 31%). They were also more likely to treat patients on public assistance (80% vs 51%) [4]. In another study comparing medical students that had participated in the International Health Fellowship Program (IHFP), 74% were practicing in primary care compared to only 43% in an age-matched cohort. Almost a quarter of them were practicing in rural areas, with 15% serving in a federally designated Health Professional Shortage Area [8]. Further studies have shown that international rotation participants were more likely than non-participants to select primary care specialties (internal medicine, family medicine, pediatrics) and work in low-income clinics [9,10].

In both developed and developing countries, there continues to be a rise in the prevalence of non-communicable diseases (NCDs) which has a significant impact on mortality and morbidity. Currently, four NCDS are responsible for half of global deaths: cardiovascular disease, diabetes mellitus, cancer and chronic respiratory diseases [11]. The rise in mortality from these NCDs has exploded in some resource limited countries by 20%-70% in the last decade [12]. Domestically, despite an increase in the total number of physicians per capita, the number of primary care providers has remained constant. There is a significant deficit in primary care providers in both rural America as well as poor urban areas in addition to the physician shortages in resource-limited settings. Hence, there remains a growing need for primary care physicians here and abroad. In addition, the emergence of public health threats in one country quickly become grave concerns globally as demonstrated with the COVID-19 pandemic. And these threats disproportionately affect those with chronic comorbid conditions who often already have barriers to adequate health care. So, can global health opportunities solve our primary care deficiencies at home and abroad thereby addressing the rise of NCDs among our patients and their detrimental consequences during a pandemic?

To determine interest in global health among third- and fourth-year US medical students and examine any correlation with their intended field of study and any impact on rank list, an anonymous electronic survey was sent to 12 US medical schools for distribution among their students. This project was approved by the West Virginia University Institutional Review Board. The 12 medical schools were chosen as comparative schools in our geographic region (West Virginia University School of Medicine, Marshall University Joan C. Edwards School of Medicine, University of Kentucky College of Medicine, University of Louisville School of Medicine, Virginia Commonwealth University School of Medicine, University of Virginia School of Medicine, Virginia Tech Carillion School of Medicine, University of Maryland School of Medicine, Georgetown University School of Medicine, University of Pittsburgh School of Medicine, The Ohio State University College of Medicine, Case Western Reserve University School of Medicine). The electronic survey included 6 questions in total, and participants had the ability to skip or not answer questions if they chose to. No identifying information was collected. The contact with these institutions was by a brief email describing the study and link to the electronic survey sent to student services of medical schools. The electronic survey was open for 6 weeks from initial email. Two reminder emails were sent out at 2 weeks and 4 weeks to try and encourage participation. These were sent to the initial contacts at institutions, and not directly to students (**Table 1**). After 6 weeks, responses were reviewed, and data collected for each question (**Table 1**). Descriptive analyses were initially conducted for each question response followed by 2×2 table analysis for calculation of odds ratios. The survey questions addressed prior or upcoming global health experiences, future career plans in terms of particular field of study, interest in primary care or specialty, and whether global health opportunities would affect their residency program rankings.

**Table 1:** Responses to online survey questionnaire from third and fourth year US medical students

**1. Have you, or will you, participate in a global health trip during your medical school training?**	Yes 51 (57%)	No 38 (43%)
**What field of medicine do you plan on practicing?**		
Internal Medicine	18 (20%)	
Family Medicine	15 (17%)	
Obstetrics/Gynecology	10 (11%)	
Medicine/Pediatrics	7 (8%)	
Pediatrics	6 (7%)	
Anesthesiology	5 (6%)	
Emergency Medicine	4 (4%)	
General Surgery	4 (4%)	
Orthopedics	4 (4%)	
Psychiatry	4 (4%)	
Otolaryngology	3 (3%)	
Neurology	2 (2%)	
Ophthalmology	2 (2%)	
Radiology	2 (2%)	
Neurosurgery	1 (1%)	
Plastic Surgery	1 (1%)	
Radiation Oncology	1 (1%)	
Unsure	1 (1%)	
**2. Do you plan on specializing in your particular field?**	Yes 62 (70%)	No 27 (30%)
3. Will, or did, global health opportunities have an impact on what residency training program you rank/ed?*	Yes 39 (44%)	No 49 (56%)
**4. If cost were not an issue and your training would not be prolonged, would you participate in an overseas global health trip if given the opportunity?**	Yes 82 (92%)	No 7 (8%)

*Only 88 responses, 1 respondent did not answer that question.

**Figure:**
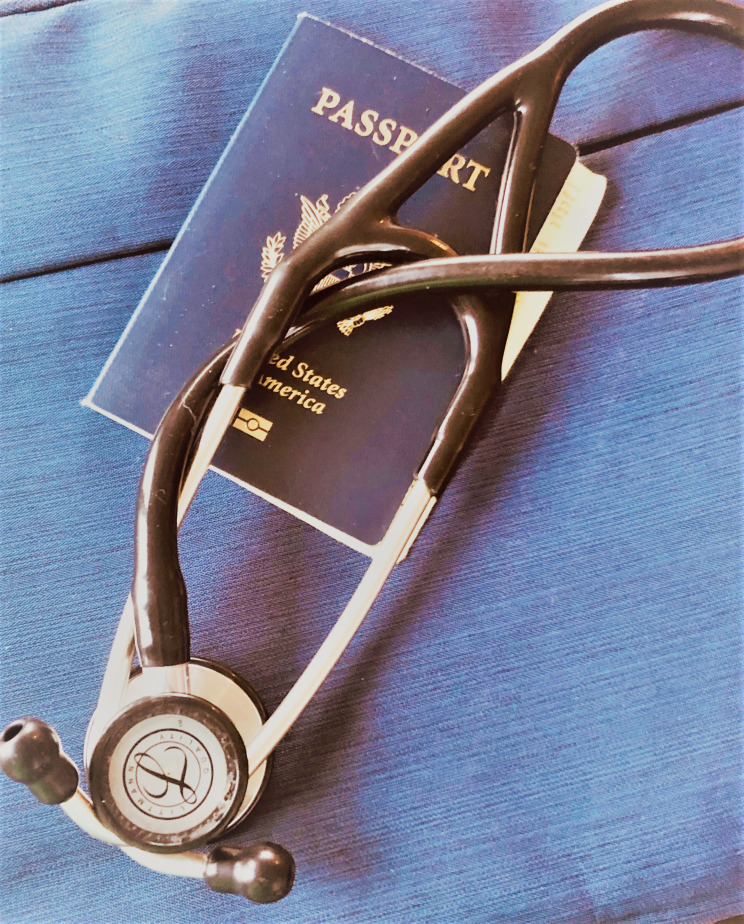
Photo: From Josephine Reece’s own collection, used with permission.

Fifty-seven percent of medical students responded with yes to having participated or will be participating in a global health elective during medical school. Almost half of respondents (46%) stated that global health opportunities would impact their ranking of residency programs. Those that answered yes to question 1 (had or will participate in a global health trip) were 15 times more likely to have global health opportunities impact their rank list (*P* < 0.001) (**Table 2**). Overall, 92% of the medical student respondents answered yes that they would participate in a global health elective if cost were not an issue and training was not prolonged.

**Table 2:** Comparison of survey answers between those that had/will participate in global health electives and those that had not/will not

	Yes (n = 51)	No (n = 38)	*P*-value
**What field of medicine do you plan on practicing?**			NA*
Internal Medicine	8 (16%)	10 (26%)	
Family Medicine	8 (16%)	7 (18%)	
Ob/Gyn	8 (16%)	2 (5%)	
Med/Peds	6 (12%)	1 (3%)	
Pediatrics	2 (4%)	4 (10%)	
Anesthesiology	2 (4%)	3 (8%)	
Emergency Medicine	3 (6%)	1 (3%)	
General Surgery	3 (6%)	1 (3%)	
Orthopedics	2 (4%)	2 (5%)	
Psychiatry	2 (4%)	2 (5%)	
Otolaryngology	0	3 (8%)	
Neurology	2 (4%)	0	
Ophthalmology	2 (4%)	0	
Radiology	0	2 (5%)	
Neurosurgery	1 (2%)	0	
Plastic Surgery	0	1 (3%)	
Radiation Oncology	1 (2%)	0	
Unsure	1 (2%)	0	
**Do you plan on specializing in your field?**			0.826
Yes	36	26	
No	15	12	
**Impact rank list?†**			<0.001
Yes	36 (71%)	5 (14%)	
No	15 (29%)	32 (86%)	
**If cost were not an issue, and training would not be prolonged, would you participate in a global health elective?**			NA
Yes	51 (100%)	31 (82%)	
No	0	7 (18%)	

*NA: not applicable due to small numbers in some categories.

†Only 88 responses, 1 respondent did not answer that question.

This study shows that global health not only remains popular for US medical students but that it can significantly impact their choice of residency training, including those pursuing IM or combine IM-pediatrics. Based on prior studies, the majority of trainees that participate in global health end up practicing in underserved areas and often in primary care [4,8]. Another study by Shull et all completed in 2008 revealed that international health elective participants were more likely than non-participants to have careers in general internal medicine post-residency (54% vs 24%, *P* < 0.01) [13,14]. By offering global health electives and/or tracks, medical institutions may attract more students to enter internal medicine or other primary care fields (family medicine and pediatrics), eroding the deficit of primary care providers for the underserved. In addition, 30% of resident participants of an internal medicine international health elective stated that the existence of global health opportunities in a residency program contributed significantly to their program preference [3,10]. APDIM’s (Association of Program Directors in Internal Medicine) annual survey sent to all internal medicine residency program directors in 2009 showed that 57% of programs offered at least a global health elective with 279 programs responding (76%). Of those that offered international rotations, 56% felt they were important to the curriculum and 46% felt they boosted recruitment. Almost a third of programs planned to expand the number of resident participants in global health electives in the future [15]. Given the positive influence that residency programs’ international opportunities have on a student’s rank list, the value of these for recruiting competitive US medical students to primary care specialties should not be overlooked.

Global health continues to be popular among medical student and resident trainees. Beyond the individual benefits to the trainee and to the international host site, offering global rotations and/or tracks can be a valuable recruiting tool for residency programs. Further longitudinal studies following physicians that have participated in global health during training are needed as much of the reported data are from the late 1990s. Do these trainees still pursue primary care careers as before, or are they following the trends of the past decade of becoming hospitalists and specialists? For general internal medicine, it may be a solution to our declining interest in primary care.
